# Effects of human demand on conservation planning for biodiversity and ecosystem services

**DOI:** 10.1111/cobi.13276

**Published:** 2019-02-27

**Authors:** Keri B. Watson, Gillian L. Galford, Laura J. Sonter, Insu Koh, Taylor H. Ricketts

**Affiliations:** ^1^ Rubenstein School of Environment and Natural Resources University of Vermont 81 Carrigan Drive Burlington VT 05405 U.S.A.; ^2^ Gund Institute for Environment University of Vermont 617 Main Street Burlington VT 05405 U.S.A.; ^3^ Department of Earth and Environmental Systems The University of the South, 735 University Avenue, Sewanee TN 37375 U.S.A.; ^4^ School of Earth and Environment Sciences The University of Queensland St Lucia QLD 4072 Australia; ^5^ Center for Biodiversity and Conservation Science The University of Queensland St Lucia QLD 4072 Australia

**Keywords:** ecosystem services, biodiversity, conservation planning, beneficiaries, demand, beneficiarios, biodiversidad, demanda, planeación de la conservación, servicios ambientales, 生态系统服务, 生物多样性, 保护规划, 受益者, 需求

## Abstract

Safeguarding ecosystem services and biodiversity is critical to achieving sustainable development. To date, ecosystem services quantification has focused on the biophysical supply of services with less emphasis on human beneficiaries (i.e., demand). Only when both occur do ecosystems benefit people, but demand may shift ecosystem service priorities toward human‐dominated landscapes that support less biodiversity. We quantified how accounting for demand affects the efficiency of conservation in capturing both human benefits and biodiversity by comparing conservation priorities identified with and without accounting for demand. We mapped supply and benefit for 3 ecosystem services (flood mitigation, crop pollination, and nature‐based recreation) by adapting existing ecosystem service models to include and exclude factors representing human demand. We then identified conservation priorities for each with the conservation planning program Marxan. Particularly for flood mitigation and crop pollination, supply served as a poor proxy for benefit because demand changed the spatial distribution of ecosystem service provision. Including demand when jointly targeting biodiversity and ecosystem service increased the efficiency of conservation efforts targeting ecosystem services without reducing biodiversity outcomes. Our results highlight the importance of incorporating demand when quantifying ecosystem services for conservation planning.

## Abstract 2

*Article impact statement*: Efforts to conserve ecosystem services are more efficient when they account for demand. Demand does not reduce biodiversity outcomes.

## Abstract (es)

Efectos de la Demanda Humana sobre la Planeación de la Conservación para la Biodiversidad y los Servicios Ambientales

**Resumen**

La salvaguardia de los servicios ambientales y de la biodiversidad es muy importante para lograr el desarrollo sustentable. A la fecha, la cuantificación de los servicios ambientales se ha enfocado en el suministro biofísico de servicios con un menor énfasis en los beneficiarios humanos (es decir, la demanda). Es sólo cuando se considera a ambos que los ecosistemas benefician a las personas, pero la demanda puede cambiar las prioridades de los servicios ambientales hacia los paisajes dominados por humanos, los cuales mantienen una menor biodiversidad. Cuantificamos cómo afecta la consideración de la demanda a la eficiencia de la conservación en la captura de los beneficios humanos y de la biodiversidad al comparar las prioridades de conservación con y sin la consideración de la demanda. Mapeamos el suministro y el beneficio para tres servicios ambientales (mitigación de inundaciones, polinización de cultivos y actividades recreativas basadas en la naturaleza) al adaptar los modelos de servicios ambientales existentes para que incluyeran y excluyeran los factores que representan la demanda humana. Después identificamos las prioridades de conservación para cada uno con el programa de planeación de la conservación Marxan. En el caso particular de la mitigación de inundaciones y la polinización de cultivos, el suministro fue un sustituto pobre para el beneficio debido a que la demanda cambió la distribución espacial de la provisión de servicios ambientales. La inclusión de la demanda cuando nos enfocamos en la biodiversidad y en los servicios ambientales como conjunto incrementó la eficiencia de los esfuerzos de conservación enfocados en los servicios ambientales sin reducir los resultados para la biodiversidad. Nuestros resultados resaltan la importancia de la incorporación de la demanda cuando se cuantifican los servicios ambientales para la planeación de la conservación.

## 摘要

保障生态系统服务及生物多样性是实现可持续发展的关键。然而, 到目前为止, 对生态系统服务的量化大多侧重于其在生物物理方面的供给, 而较少关注受益的人类 (即人类需求) 。只有同时满足以上两点, 生态系统才能造福人类, 但人类需求可能会将生态系统服务的保护重点转向那些只能支持较少生物多样性存在的人类主导的景观。通过比较有无考虑人类需求时的保护优先事项, 我们对其如何影响保护中的人类福祉及生物多样性保护成效进行了量化分析。我们在现有的生态系统服务模型中分别加入和去除代表人类需求的因素, 绘制出三种生态系统服务 (防洪、作物传粉、自然娱乐) 的供给和效益情景图。然后, 我们用保护规划软件 Marxan 确定了每种生态系统服务的保护优先事项。结果表明, 生态系统服务的供给有时并不能很好地代表效益, 特别是在防洪和作物传粉方面, 这是因为需求改变了生态系统服务供给的空间分布。以生物多样性及生态系统服务的保护为目标时, 考虑人类需求可以在不损害生物多样性保护成果的前提下, 提高生态系统服务保护工作的成效。我们的结果强调凸显了保护规划中量化生态系统服务时, 将人类需求纳入其中的重要意义。**【翻译: 胡怡思; 审校: 聂永刚】**

## Introduction

Ecosystem services (ESs) are the direct and indirect contributions of ecosystems to human well‐being. Environmental degradation has decreased the capacity of ecosystems to support biodiversity and to provide nonmarket ESs (Millennium Ecosystem Assessment 2005). Maintaining ESs while safeguarding biological diversity is essential to achieving sustainable development (ICSU 2015), yet the degree to which these 2 goals can be achieved through the same actions is unclear.

Conservation organizations increasingly target ESs and biodiversity (Ruckelshaus et al. 2013; Mace 2014; Guerry et al. 2015) under the often implicit assumption that land conservation efforts can simultaneously achieve biodiversity and ES goals. For instance, The Nature Conservancy (TNC) and Conservation International have each revised their mission statements to include explicit reference to ESs (Doak et al. 2014), and a survey of 60 TNC projects showed that 34 (57%) explicitly target ESs (Goldman et al. 2008). However, allocating resources toward ESs may reduce the resources available to conserve biodiversity (McCauley 2006; Luck et al. 2012; Reyers et al. 2012) given limited conservation budgets. The severity of this trade‐off hinges on the spatial overlap of priorities for biodiversity and ESs (Chan et al. 2006; Withey et al. 2012; Kovacs et al. 2013). For example, targeting emissions reductions from avoided deforestation may undermine biodiversity conservation in Indonesia because priorities for each do not spatially coincide (Paoli et al. 2010). The spatial concordance between biodiversity and specific ESs depends on a number of factors, such as whether or not they are linked via a functional relationship (Kremen 2005; Luck et al. 2009) and spatial scale (Cimon‐Morin et al. 2013) and the time scale considered. The metric used to quantify ESs may also affect this relationship (Ricketts et al. 2016).

Quantifying ESs involves both supply (i.e., ecosystem functions with the potential to benefit people) and demand (desired amount of human consumption of that supply, which depends on peoples’ desire for and access to ESs) (Fisher et al. 2009; Tallis et al. 2012; Yahdjian et al. 2015). Benefits to people arise from the interaction of supply and demand. For example, riparian wetlands can dissipate flood peaks but this function only provides a benefit if there are people downstream at risk of flooding (Watson et al. 2016). Beneficiaries of ESs vary in their preferences and vulnerability and thus the extent to which they value those benefits. For example, the value of avoided flooding may be highest for uninsured and poor people (Brouwer et al. 2007; Arkema et al. 2013). Accounting for demand when measuring benefits, and beneficiary preferences when measuring value, are current best practices in ES science (Tallis et al. 2012). We focused on the former: the interaction of supply and demand to produce benefit.

Supply is sometimes used as a proxy for benefit because the data and models to quantify supply are more readily available (Egoh et al. 2009; Maes et al. 2012; Lin et al. 2017). As a result, conservation projects may protect supply in places where demand is low or absent, thus capturing benefits inefficiently. Efficiently targeting conservation to safeguard ESs requires understanding the spatial relationship between where ESs are supplied, where people exhibit demand for ESs, and how ES supply is connected to ES demand to produce benefits (Villamagna et al. 2013; Bagstad et al. 2014; Schröter et al. 2014; Serna‐Chavez et al. 2014).

Incorporating demand may exacerbate trade‐offs between biodiversity and ESs if conservation efforts targeting benefits safeguard less biodiversity than efforts targeting supply (Balvanera et al. 2014; Ricketts et al. 2016). Benefit may be less tightly linked to biodiversity than supply (Cardinale et al. 2012) because it is modified by demand; the human focus of demand may weaken the functional link (Mitchell et al. 2013) and the spatial concordance (Reyers et al. 2012; Ricketts et al. 2016) between ESs and biodiversity.

Few efforts explicitly quantify the consequences of including demand into conservation efforts to safeguard ESs and biodiversity (Wolff et al. 2015; Verhagen et al. 2016). We aimed to quantify differences between supply and benefit within the context of conservation planning. We addressed 3 questions: How does incorporating demand shift the spatial distribution of benefits relative to supply? How much benefit is captured by conservation efforts that target supply? How do efforts targeting supply and benefit compare in terms of their biodiversity outcomes?

## Methods

We mapped 3 ESs in terms of supply and in terms of benefit (interaction of supply and demand). We simulated optimal conservation networks for each of supply, benefit, and biodiversity with the optimization program Marxan (Ball et al. 2009). We then compared the effectiveness of each network in capturing biodiversity and benefits.

### Quantifying ESs

We quantified supply, demand, and benefit for 3 locally important ESs: flood mitigation, nature‐based recreation, and crop pollination across Vermont, U.S.A. (Table 1). Vermont depends heavily on local food and tourism sectors (Sonter et al. 2016) and has been affected recently by major floods (Watson et al. 2016). Our landscape comprised 4462 hexagonal polygons, each 5.85 km^2^ in area, approximately the average size of existing conserved lands in Vermont (mean = 6.7 km^2^, median = 10.1 km^2^) (The Nature Conservancy 2012). Analyses for each ES were performed at different spatial scales, and we aggregated supply and benefit to the hexagon scale as the sum of contained pixels.

**Table 1:** Ecosystem service supply and benefit as defined through our analyses

	Supply	Benefit
Flood mitigation	retention of quick flow by natural ecosystems relative to pasture, the dominant anthropogenic landscape	retention of quick flow weighted by the number of downstream structures in a flood risk area
Nature‐based recreation	visitation by recreants as a function of natural landscape features	visitation by recreants as a function of landscape features and surrounding population density
Crop pollination	wild bee abundance	wild bees foraging on pollinator‐dependent crops

Flood outcomes are determined by the quantity and timing of water entering river channels and the hydraulic properties of a river's channel and floodplain. Quick flow is the portion of water that moves quickly to a channel via surface runoff or interflow and is the portion of runoff likely to generate a flood. We quantified supply as the retention of quick flow by natural land‐cover types relative to pasturelands (dominant anthropogenic land‐cover class in our study area) with the InVEST model for monthly water yield (Sharp et al. 2014; Guswa et al. 2017) (Supporting Information). Channel and floodplain effects are beyond the scope of this work.

We defined demand as the number of downstream buildings at risk of flooding. We overlaid spatial data sets of buildings (E911 Board 2013) and floodplain areas (Sangwan & Merwade 2015) in ArcGIS (ESRI 2012) to identify at‐risk buildings. We used the InVest DelinateIT model (Sharp et al. 2014) to delineate the watershed draining to each floodplain polygon that contained buildings. We assigned a demand score to each pixel in which each structure equated to 1 unit of demand distributed evenly to all of the pixels in its upstream drainage so that each pixel received a demand score that was the sum of the demand from all downstream structures. Dividing demand equally among all upstream pixels was a simplifying but necessary assumption. The portion of a watershed that is most important in contributing to, or dissipating, a flood peak is highly dependent on the duration, spatial distribution, and intensity of a particular rainfall event. A similar conceptualization of demand for flood mitigation is established in the literature (Sturck et al. 2014).

We standardized supply and demand on a scale of 0–1 and calculated benefit as the product of supply and demand. This multiplicative effect represented the interaction of supply and demand to produce benefit; if either supply or demand was 0, benefit was also 0. By taking the unweighted product of supply and demand, we assumed both were equally important in determining benefit. Our results were insensitive to this assumption (Supporting Information). All calculations were performed at a 30‐m resolution.

We mapped the supply and benefit of nature‐based recreation with a model previously developed for Vermont (Sonter et al. 2016). This model was calibrated with empirical data on visitation to Vermont state parks and predicted visitation to other conserved areas in Vermont based on geotagged photographs uploaded to the website Flickr. Ten predictor variables were tested; 7 were found to significantly explain visitation rates. We predicted the number of visits that each hexagon would receive if it were to be conserved. To map supply, we predicted visits based on the 4 significant variables related to natural landscape features (average forest cover, average slope, number of opportunities to swim [i.e., sites with accessible lakes or beaches] and number of opportunities to ski [i.e. sites with accessible ski trails]) and the average value of 2 significant variables related to development as a conserved land (trail density and publicly accessible land area [i.e. conserved public land]). To map benefits, we predicted visits based on the additional explanatory variable that represented demand—population density within 25 km of each hexagon. A 25‐km radius was used in the original model because this was the average distance between conserved areas in Vermont and it significantly explained visitation across the state. Visitation was standardized on a scale of 0 to 1 for both supply and benefit.

We used Koh et al. (2016) estimates of wild bee abundance as our measure of supply. Koh et al. (2016) used a published model of wild bee abundance (Lonsdorf et al. 2009), in which bee abundance depends on nesting sites and floral resources within an average foraging distance of 670 m. The average nesting and floral resource availability of 45 representative land‐cover types (32 crop and 13 noncrop categories) were parameterized for 4 different bee nesting guilds and 3 foraging seasons by collecting and validating experts’ opinion. To estimate demand for crop pollination, we used Koh et al.’s (2016) map of pollinator dependent crops, which weighted each crop within the cropland data layer (USDA‐NASS 2013) according to published pollinator dependency rates. We used this map to calculate a distance‐weighted sum of pollinator dependent crops in the neighborhood around each cell. The neighborhood was defined based on an exponential decay function describing the foraging distance of bees, where the average forage distance was 670 m. We standardized indices of both supply and demand to a scale of 0–1 and defined benefit as the product of these 2 indices. This benefit index is high for pixels with high bee abundance (supply) that are surrounded by pollinator‐dependent crops (demand).

### Quantifying Biodiversity

We defined *biodiversity* as the variety of life traditionally prioritized by conservation actions—a select part of biodiversity rather than diversity per se. As the component of overall biological diversity that is most valued by people, this can also be thought of as existence value. We used BioFinder, an existing statewide map of conservation priorities used by organizations such as the Vermont Agency of Natural Resources and the Vermont Land Trust (Austin et al. 2013), to measure biodiversity. This data set is the best available representation of how conservation prioritization for biodiversity is being put into practice in Vermont. BioFinder identifies “high priority ecosystems, natural communities, habitats, and species” as the weighted sum of 21 data sets, including landscape‐scale (e.g., riparian wildlife connectivity, physical landscape diversity) and community‐scale (e.g., rare species, rare natural communities) indicators (Austin et al. 2013).

We tested the sensitivity of our results to BioFinder by identifying conservation priorities based on 234 different vertebrate species ranges from the USGS GAP data set (U.S. Geological Survey Gap Analysis Program 2011). Conservation priorities based on BioFinder and priorities based on vertebrate species did not differ substantially (Supporting Information).

### Costs of Conservation

We used land value to approximate the relative costs of conservation. For roughly 50% of our study area, public tax records of property values could be associated with digitized parcel maps. We estimated unknown land values with a generalized additive model with socioeconomic predictors and a spline smoother for spatial location (Bivand 2008) because land values are spatially correlated. Distance to cities, median household income, predominant land cover, density of built structures, road density, and the presence of urban centers explained over 50% of the variation in log‐transformed land costs (*r* ^2^ = 0.532, df = 16, all coefficients significant at *p* < 0.05). The spline term significantly improved the model (approximate *p* < 2.2 × 10^−16^, all coefficients significant at *p* < 0.05) (Supporting Information). We used the predicted log‐transformed land cost as an index of relative costs of conservation to minimize the effect of high‐value developed areas on the mean value at a hexagon scale—these areas are not characteristic of protected areas.

### Comparison of Supply and Benefit

To determine how demand affects ESs, we compared the density and spatial distributions of supply and benefit. We measured density distributions as kernel density with the geom_density function of the “ggplot2” package in R. We compared density distributions of supply and benefit with a 2‐sided Kolmogorov–Smirnov test. We tested cross‐autocorrelation of supply and benefit in space with the centered Mantel statistic as implemented in R's ncf package (Bjornstad 2009).

### Identifying Conservation Priority Areas

We identified conservation priority areas under 4 targeting strategies: supply, benefit (both single‐factor optimization), supply and biodiversity, and benefit and biodiversity (both multifactor optimization). We also performed an optimization for biodiversity alone as a control. Identifying joint spatial priorities for biodiversity and ESs better illustrates opportunities to achieve both targets than assessing their spatial correlation. Correlations reflect similarities between places with both low and high value, but only high‐value areas are relevant in the context of spatial planning. Even if correlation overall is low, there may still be locations that efficiently conserve biodiversity and ESs.

We used Marxan (Ball et al. 2009) to identify priority areas for each ES under each of the 4 targeting strategies and for biodiversity. Marxan approximates optimal conserved lands networks via a simulated annealing algorithm given the value and cost of each unit of analysis by minimizing the objective function:

(1) ObjFun  min = land  cost (x,y)+λ( protection  target - protection  achieved )i+ cost  constraint ,

where land cost is the sum of land‐cost index for all hexagons within the selected priority areas; *i* is the targeted conservation features (in our case biodiversity, supply, or benefit); protection target is the target amount of a conservation feature that the optimization seeks to achieve; protection achieved is the amount of a conservation feature held within the selected priority areas; λ is the species penalty factor for missing a conservation feature's protection target (essentially a weighting of the importance of each conservation feature); and cost constraint is a penalty for exceeding a user‐defined cost constraint. We set equal weights for biodiversity and ESs and set the cost constraint penalty high enough such that the solution never exceeded our constraint.

We set a cost constraint that allowed approximately 15% of the landscape to be selected and protection targets that were impossible to reach given that constraint (50% of statewide supply, benefit, or biodiversity). Optimal solutions never exceeded the cost threshold and maximized the protection of conservation features within that constraint (a maximum coverage problem).

We performed 500 runs for each simulation and used the best solutions as our priority areas (Ball et al. 2009). This process identified priority areas for ES and biodiversity as though we redesigned conserved lands today based on these criteria and set aside approximately the same amount of land area that is currently protected.

To assess how well different targeting strategies captured the best places for supply, benefit, and biodiversity, we performed a post hoc test in which we calculated the return on investment (ROI) of each hexagon in terms of biodiversity, supply, and benefit, as the amount of each per unit cost. We then standardized ROI as a percentile rank.

## Results

### Demand and ES

**Figure 1:**
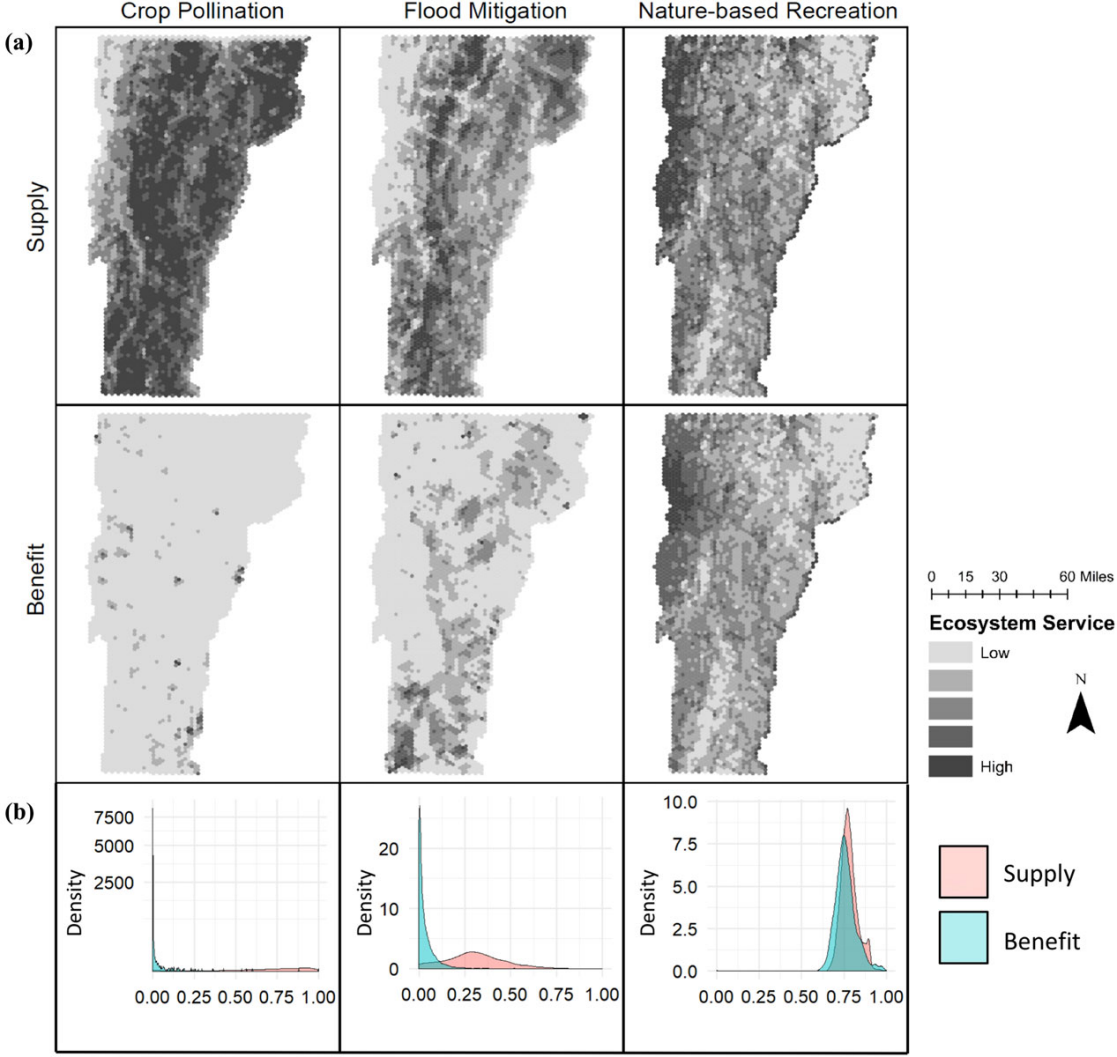
(a) Spatial and (b) density distribution of ecosystem service supply and benefit for crop pollination, flood mitigation, and nature‐based recreation in Vermont (maps: the darker the shading the higher the level of supply or benefit; 5 shades categorized with the natural breaks function in ArcMap; graphs: density distributions are a smoothed version of a histogram and illustrate the continuous nature of supply and benefit indices; density, relative density of values within a range such that the area under the entire curve equals 1).

Demand shifted the spatial distribution of each ES (Fig. 1a), although supply and benefit were highly correlated for nature‐based recreation (Mantel statistic for crop pollination *r*_s_ = −0.08, flood mitigation *r*_s_ = 0.26, nature‐based recreation *r*_s_ = 0.95, *p* < 2.2 × 10^−16^ e^−16^ in all cases) (Fig. 1). Density distributions also differed between supply and benefit for all 3 ESs, but this difference was much smaller for nature‐based recreation (Kolmogorov–Smirnov test for crop pollination *D* = 0.95, flood mitigation *D* = 0.80, nature‐based recreation *D* = 0.20, *p* < 2.2 × 10^−16^ in all cases) (Fig. 1b). Priority areas were similar for supply and benefit of nature‐based recreation, but noticeably different for flood mitigation and crop pollination (Fig. 1).

### Supply as a Proxy For Benefit

**Figure 2:**
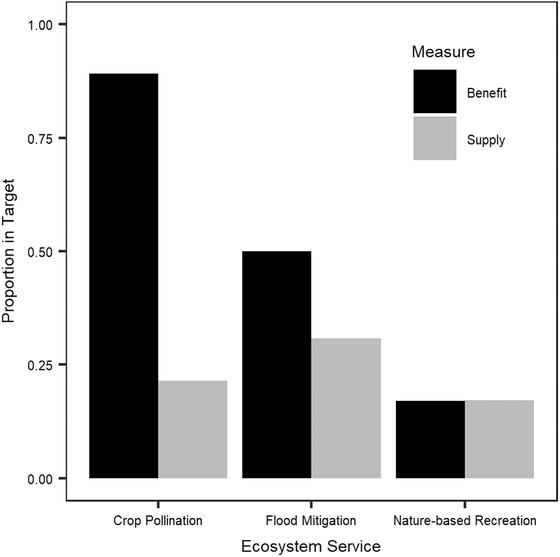
Proportion of ecosystem service (ES) benefit in priority areas targeting ES supply and benefit for crop pollination, flood mitigation, and nature‐based recreation.

For crop pollination and flood mitigation, priority areas targeting benefit contained more benefit than priority areas targeting supply (Fig. 2). Although this is an expected outcome of our optimization, the size of the difference between targeting supply versus benefit varies across ESs. Priority areas targeting benefit took up 12.2% and 7.0% of the landscape while capturing 50% and 89% of benefit for flood mitigation and crop pollination, respectively, but for nature‐based recreation, priority areas targeting supply and benefit both captured approximately 17% of benefit.

### Biodiversity Outcomes

**Figure 3:**
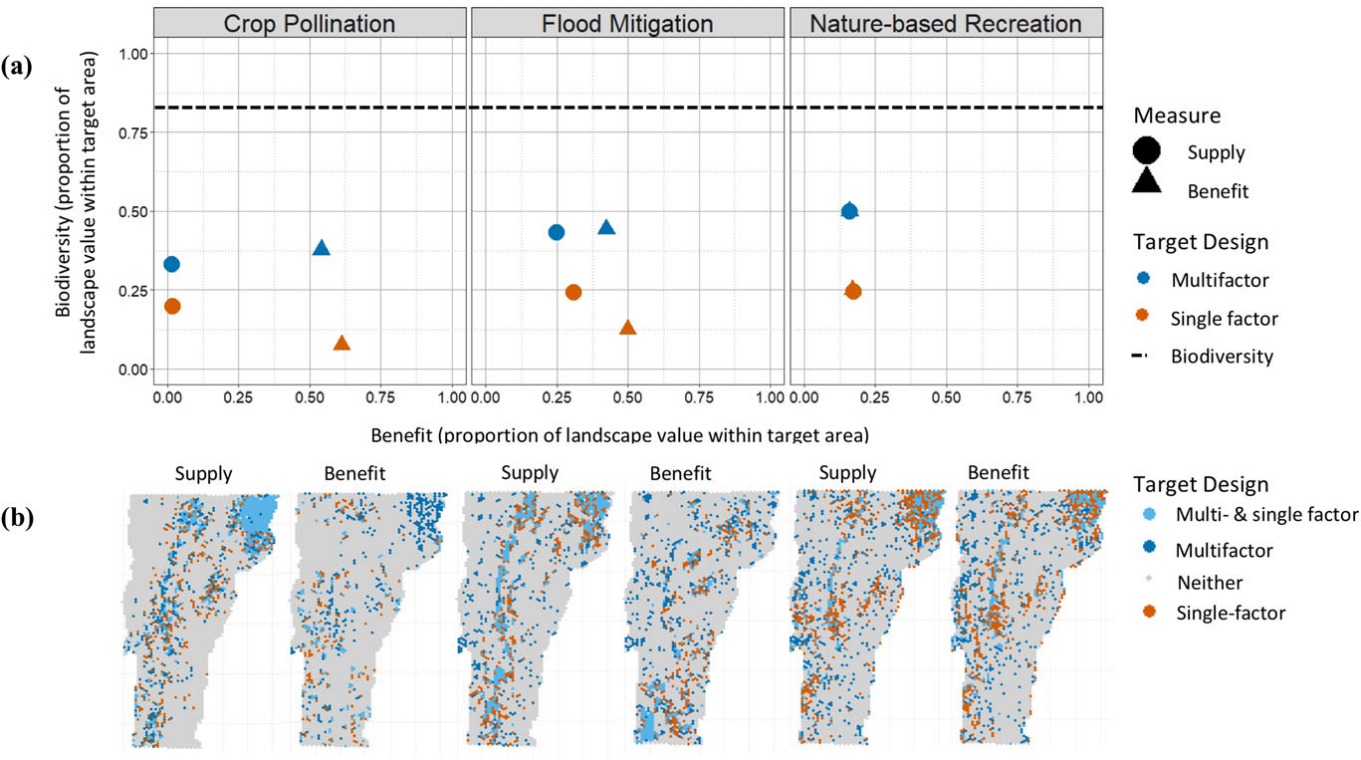
(a) Ecosystem service (ES) and biodiversity in priority areas for single‐factor and multifactor optimization strategies and (b) maps of ecosystem service priority areas (orange, single‐factor optimizations for either ES supply or benefit; dark blue, multifactor optimizations for supply or benefit jointly with biodiversity; light blue, locations within the conservation optimization for both multifactor and single‐factor optimizations; dashed line, level of biodiversity captured by a single‐factor optimization for biodiversity).

Across ESs single‐factor strategies contained on average 29% of the biodiversity that could be captured by targeting biodiversity alone. Priority areas for benefit and priority areas for supply captured similar amounts of biodiversity for crop pollination and nature‐based recreation. Flood mitigation priorities for benefit captured less biodiversity than priorities for supply (Fig. 3). Multifactor optimization improved biodiversity outcomes for all ESs. Across the 6 possible comparisons (3 ESs, supply, and benefit), targeting ES and biodiversity jointly increased biodiversity by 149% on average and reduced ESs by 13%.

**Figure 4:**
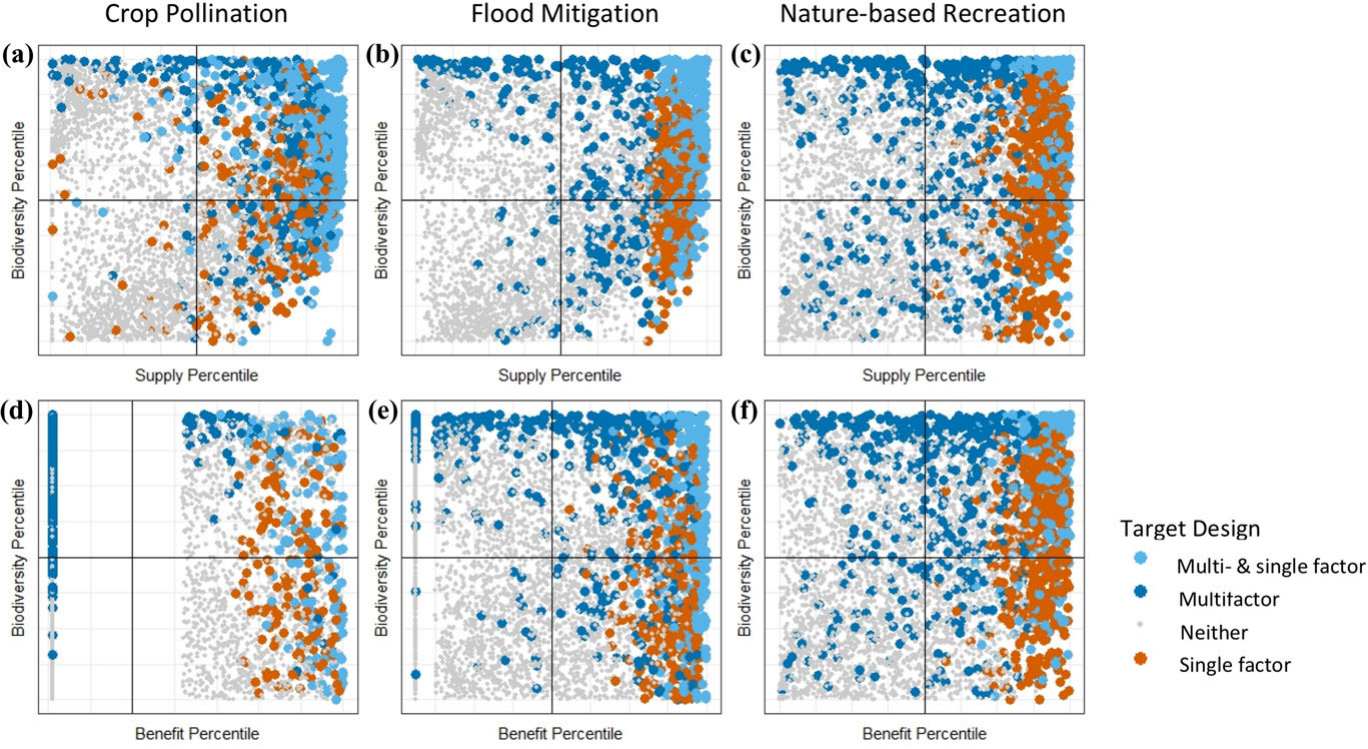
Return on investment for biodiversity and ecosystem services of units of analysis selected under different optimizations. Panels depict the percentile rank of each hexagon for ecosystem service (supply or benefit) on the x‐axis and biodiversity on the y‐axis (each axis is therefore scaled from 0 to 100) (return on investment, ratio of ecosystem service or biodiversity to conservation cost; top panels, supply; bottom panels, benefit).

Multifactor optimization shifted the spatial distribution of priority areas relative to single‐factor optimizations (Fig. 3). For supply for all ESs and for benefit for nature‐based recreation, multifactor optimizations tended to select priority areas that ranked in the upper 50th percentile for both criteria (Fig. 4a–c, f). Flood mitigation and crop pollination multifactor optimizations for benefit included places that were important for both biodiversity and benefit and places important for biodiversity even when they contained very little benefit (Fig. 4d, e).

## Discussion

Incorporating demand increased the efficiency of conservation efforts targeting ESs without reducing biodiversity outcomes. Demand shifted the spatial distribution of benefit relative to supply such that supply served as a poor proxy for benefit. Targeting supply did not capture more biodiversity than targeting benefit. Single‐factor priority areas captured little biodiversity, and joint targeting greatly improved biodiversity outcomes with small consequences for benefit.

Human demand for ESs shifted benefits relative to supply in 2 ways: concentration and spatial shift. First, For flood mitigation and crop pollination, demand concentrated benefit such that small areas of supply provided significant benefits, but most supply provided little benefit. For nature‐based recreation, demand shifted benefit toward population centers without altering the density distribution of benefit relative to supply.

Demand concentrates benefit if benefits are greater nearby demand or in small service sheds (Tallis et al. 2012; Mandle et al. 2015). The flow of crop pollination is limited by the flight distance of bees; thus, supply provides benefit only when it is near demand. When service sheds vary in size (e.g., flood mitigation), the marginal impact of losing a given quantity of supply will be highest in small service sheds that have less supply to start with (Fisher et al. 2008).

Spatial shifts occurred when ES flows connected all sources of supply to at least 1 source of demand. All sources of supply provided benefit, although benefit shifted toward sources of demand. Recreants in Vermont travel to obtain recreational opportunities, which are generally available within a 2‐hour drive (Sonter et al. 2016); thus, any location that supplies nature‐based recreation provides benefit. At its extreme, for some ESs all supply may provide equal benefit, for example, carbon sequestered in forests affects climate globally (Cramer et al. 2004; Bonan 2008).

When demand concentrates benefit, efforts that prioritize supply are less efficient in safeguarding benefits. However, when demand causes a spatial shift, supply may serve as an acceptable proxy for benefit. Although further study is needed to test the generalizability of these 2 cases, our results indicated the spatial and density distributions of supply and demand can inform decisions about when incorporating demand is critical (concentration) and when doing so will achieve smaller efficiency gains (spatial shift).

Efforts that target ES without considering biodiversity are unlikely to provide high levels of biodiversity regardless of the ES measure used (supply or benefit). Many conservation organizations target ES in addition to (not instead of) biodiversity (Reyers et al. 2012). This is represented by our multifactor optimizations, which double biodiversity outcomes relative to single‐factor optimizations with minimal impact on ESs. There is still a biodiversity trade‐off in targeting ESs. Equally weighting ESs and biodiversity in multifactor optimization caused a larger trade‐off for biodiversity (31% reduction relative to a single‐factor optimization for biodiversity) than ES (13% reduction relative to single‐factor optimizations for ES).

Human demand is the component of ES that makes them distinct from other ecological measures (Fisher et al. 2009) and thus is the source of concern that ES will shift conservation priorities towards human‐dominated landscapes (Reyers et al. 2012). For flood mitigation and crop pollination, the single‐factor optimization for supply captured more biodiversity than the single‐factor optimization for benefit. All multifactor optimizations for benefit captured roughly the same amount of biodiversity as the comparable optimization for supply. Because benefit and supply are distinguished by incorporating or omitting demand, our results indicated that, although demand is critical in efficiently capturing benefits to people, including it in conservation plans does not necessarily reduce biodiversity outcomes.

For flood mitigation and crop pollination, the biodiversity gains of multifactor optimizations were not achieved by conserving places important for both biodiversity and benefit. Because demand concentrated benefit, actions taken on a small portion of the landscape represented disproportionately large benefits for people, and the remaining budget was used to conserve high biodiversity areas regardless of their importance for ESs. Thus, both can be protected even when unit‐by‐unit co‐occurrence is low. While this result is sensitive to the budget constraint, our 15% constraint is reasonable in many conservation contexts. This opportunity arises as a result of demand concentrating benefit and occurs even when benefit occurs in places that are less important for biodiversity. Thus, incorporating demand may decrease, rather than exacerbate, trade‐offs between ES and biodiversity.

These findings have direct consequences for conservation practice. Organizations seeking to safeguard ES are likely to be more effective in doing so is they consider demand, but should not assume their actions will have large biodiversity co‐benefits unless they explicitly seek them out. Organizations engaging with ES as a means of supporting biodiversity conservation face inherent trade‐offs when splitting budgets between 2 goals. Multifactor optimization alleviates these trade‐offs, and incorporating demand may do so as well.

Several limitations in our analysis remain. Future researchers should test whether our findings hold for other ESs in other regions and where biodiversity priorities have been determined differently (e.g., where different components of biodiversity are valued and targeted by conservation organizations). We ignored differences in how groups of beneficiaries may value benefits to illustrate the effect of accounting for or omitting demand altogether. For instance, we could have weighted structures within floodplains according to expected flooding frequency, vulnerability to flooding, or economic value (though the latter unfairly implies wealthier homes are more valuable for human well‐being). We faced methodological challenges when integrating supply and demand to determine benefit. For nature‐based recreation, we used regression to determine the relative importance of supply and demand in determining benefit. For flood mitigation, the relative importance of supply and demand in benefit was unknown; we assumed each component contributed equally and calculated benefit as their unweighted product. Finally, we assessed the relationship between biodiversity and individual ESs, whereas biodiversity likely relates to suites of ESs (Duffy 2009). Optimizing all 3 ES simultaneously, rather than individual ES, may capture more biodiversity.

Nature underpins human well‐being, and conservation can help advance a broad range of sustainable development goals (ICSU 2015). While it is widely acknowledged that these goals are interdependent, opportunities to work towards several goals at once are less clear. Our findings indicate that land conservation is one such opportunity; it can reduce biodiversity loss while achieving other human wellbeing outcomes. Although our results are specific to the location and ESs we focused on, they illustrate how incorporating demand, the human‐centered component of ecosystem services, into ES priority setting can significantly augment benefits to people without equivalent drawbacks for biodiversity. Furthermore, ignoring demand (i.e., using supply as a proxy for benefit) can result in significant missed opportunities. Thus the distinction between the biophysical supply of services and the benefits of ES to people is not just a theoretical and semantic issue, but rather a matter of critical importance for the outcomes of conservation in practice.

## Supporting information

Details on the InVEST Seasonal Water Yield model (Appendix S1), a sensitivity analyses of flood‐mitigation supply results to including winter months in the model (Appendix S2) and of our flood‐mitigation service results to the assumption that supply and demand are equally important in determining benefit (Appendix S3), an explanation of modeled land value as a proxy for conservation cost (Appendix S4), maps of demand for each ES (Appendix S5), a comparison of BioFinder to species‐based conservation prioritization (Appendix S6), and a comparison of single‐factor optimizations to hotpots selected based on ROI (Appendix S7) are available online. The authors are solely responsible for the content and functionality of these materials. Shapefiles of ecosystem service supply, demand, and benefit, and best conservation networks for each are freely available from K.W.’s FigShare account: https://figshare.com/authors/Keri_Watson/6259679. Queries (other than the absence of the material) should be directed to the corresponding author.
