# Supplementary material for: Effects of human demand on conservation planning for biodiversity and ecosystem services
Source: Conserv Biol. 2019 Feb 27;33(4):942–52. doi: 10.1111/cobi.13276 (PMC6850574; doi:10.1111/cobi.13276)

## **Supporting Information**

**Appendix S1:**

The InVEST model estimates quick-flow as the portion of runoff with a residence time of hours to days, as a function of soil type, topography, precipitation, and land-cover. It adapts a curve number approach [(Mockus 2004)](https://paperpile.com/c/sggG3z/ME9f8) to a pixel resolution and a monthly time step, and has been shown to effectively approximate the proportion of rainfall that runs off as quick-flow across the continental U.S. [(Guswa et al. 2017)](https://paperpile.com/c/sggG3z/QuTuM), and New England [(Blumstein & Thompson 2015)](https://paperpile.com/c/sggG3z/y8yIj). We parameterized the model (Supporting Information) to represent the generation of quick-flow from rainfall events onto saturated soils (ARCIII conditions, [(Mockus & Hjelmfelt 2004)](https://paperpile.com/c/sggG3z/6l0Lp)), and then produced a supply index by calculating standardized quick-flow for each pixel on a zero to one scale. The curve number approach is not appropriate for snow. Historically, Vermont has not received rainfall in winter months, but in recent years rainfall has occurred year round, although winter months remain snow dominated. We calculated our supply index with and without winter months included. The resulting indices were essentially identical, so in the subsequent analyses we use the 12 month supply index.

Input data and parameterization of the InVest Seasonal Water Yield model:

| **Model Input** | **Data Source** |
| --- | --- |
| Average monthly precipitation | We downloaded 30 year monthly precipitation normals from the PRISM Climate Group for the period 1981- 2010 [(PRISM Climate Group 2012)](https://paperpile.com/c/sggG3z/hicsz). |
| Monthly reference evapotranspiration | Reference evapotranspiration data was derived from CCIGAR’s globally available data on potential evapotranspiration [(Trabucco & Zomer 2009)](https://paperpile.com/c/sggG3z/ZxMrp). |
| Land-cover | Land-cover data was derived from the national landcover dataset (2011) [(Homer et al. 2015)](https://paperpile.com/c/sggG3z/XciLq). |
| Hydrologic soil group | Hydrologic soil group obtained from SSURGO soils data [(USDA Natural Resources Conservation Service n.d.)](https://paperpile.com/c/sggG3z/71SFa). No data values were assigned the value C because this hydrologic group was the most common within Vermont (comprised a larger total area than any other hydro-group). Open water pixels were assigned to group D. |
| Curve numbers for each soil type/land-cover combination | We adopted standard curve numbers for each NLCD land-cover class and soil hydrologic group under wetter antecedent runoff conditions (ARC III) [(Victor Mockus 2004)](https://paperpile.com/c/sggG3z/87B2b) as follows:  **NLCD classification - NEH Cover description treatment** [(Mockus 2004)](https://paperpile.com/c/sggG3z/ME9f8)  Developed open space - Open space, good condition  Developed low intensity - Residential districts: lot size 1/4 acre  Developed, medium intensity - Residential districts: lot size 1/8 acre or less  Developed, high intensity - Urban districts: commercial and business  Barren land - Bare soil  Deciduous forest - Woods good, condition  Evergreen forest - Woods good, condition  Mixed forest - Woods good, condition  Shrub scrub - Brush-forbes-grass mixture, good condition  Herbaceous - Brush-forbes-grass mixture, good condition  Hay/pasture - Pasture, grassland, or range-continuous forage for grazing, good condition  Cultivated crops - Straight row- good condition  Woody wetlands - Woods, good condition  Emergent herbaceous wetlands - Woods, good condition |

**Appendix S2:** The sensitivity of our flood mitigation supply results to including winter months in the model.


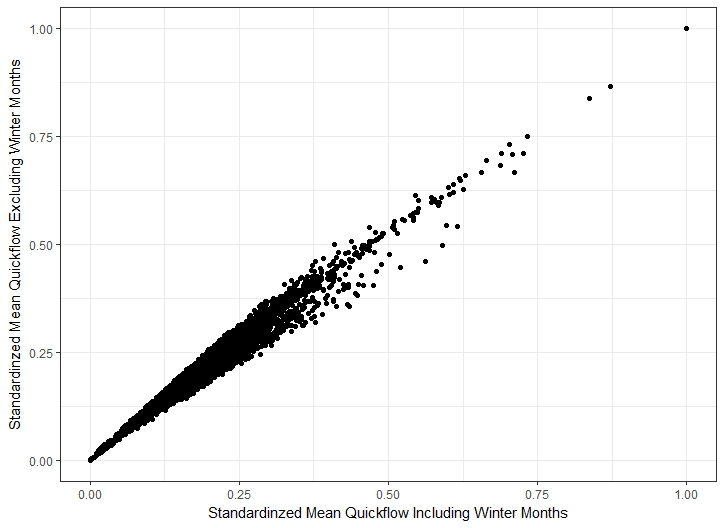


**Appendix S3:** The sensitivity of our flood mitigation service results to the assumption that supply and demand are equally important in determining benefit. When assigned demand is one half (grey) and one tenth (black) the weight of supply, the major conclusions about the differences between supply and benefit, and the biodiversity and benefit value of priority areas hold.


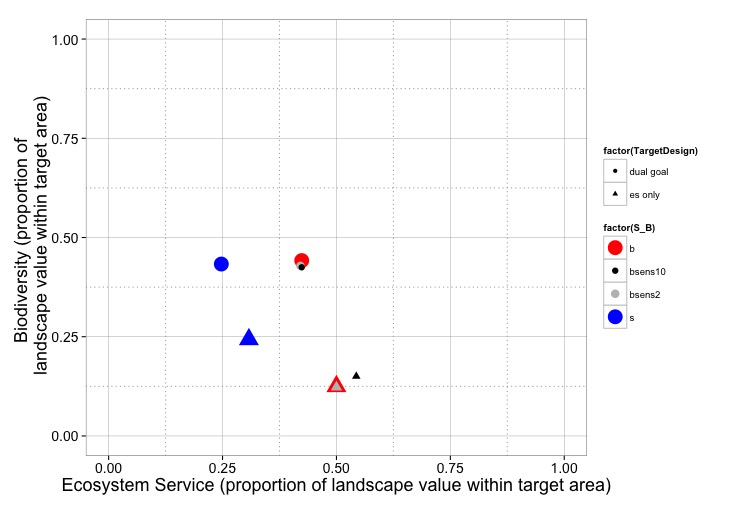


**Appendix S4:** A) Predicted land value plotted against land value for all hexagons where land value data was available. We predicted land value using a generalized additive model with a spatial spline smoother (r2=0.584, df=44.04). B) The resulting distribution of log(Land Value), was used as an approximation of conservation costs. We use log-transformed costs because the extremely large variation in untransformed land costs likely does not apply to conservation investments; it is driven by the density and prevalence of developed land in each hexagon, which are unlikely to be targeted by conservation efforts. Further, untransformed land costs varied much more widely than did supply and benefit, and their variation otherwise outweighed differences between the two when selecting optimal conservation priorities.


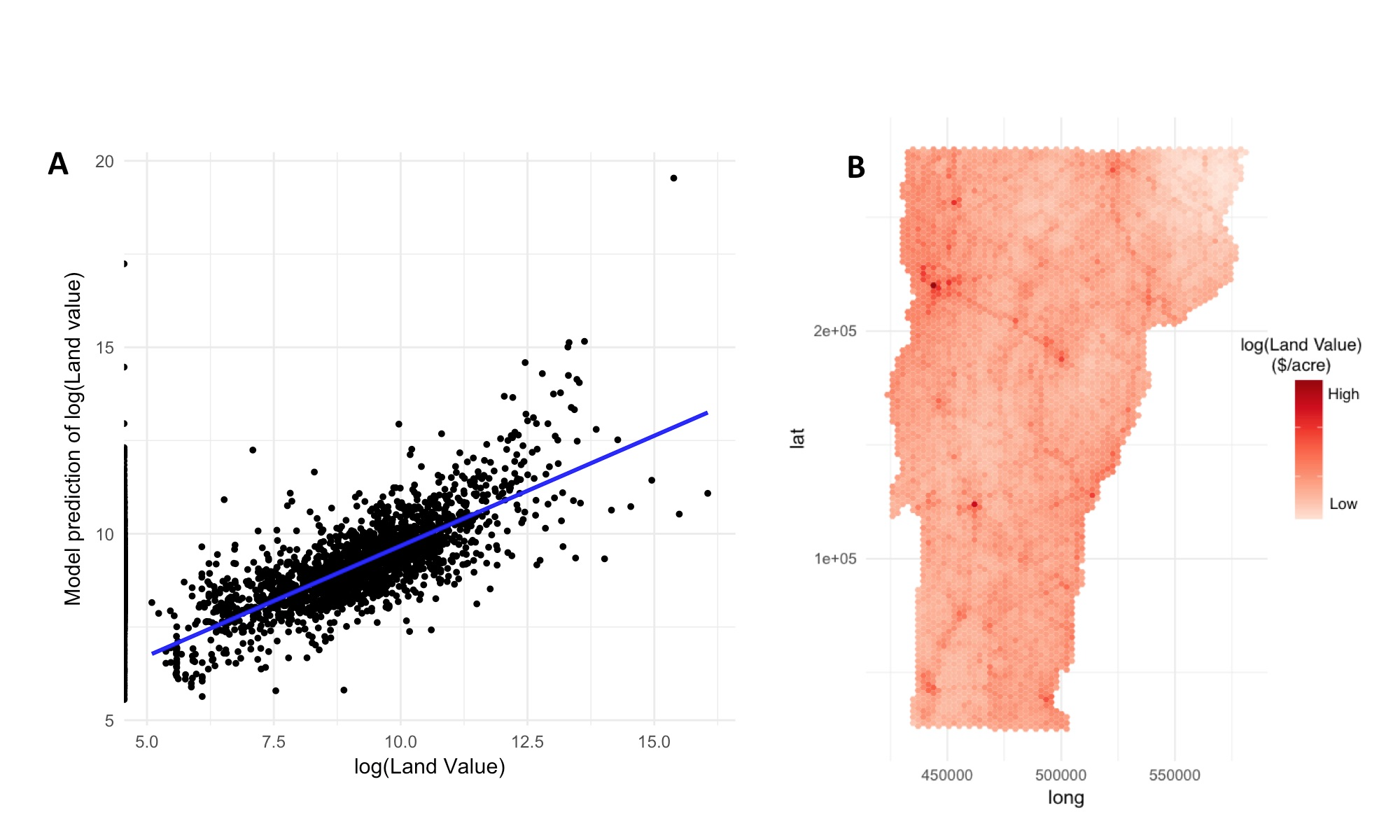


**Appendix S5:** Maps of demand for each ecosystem service.


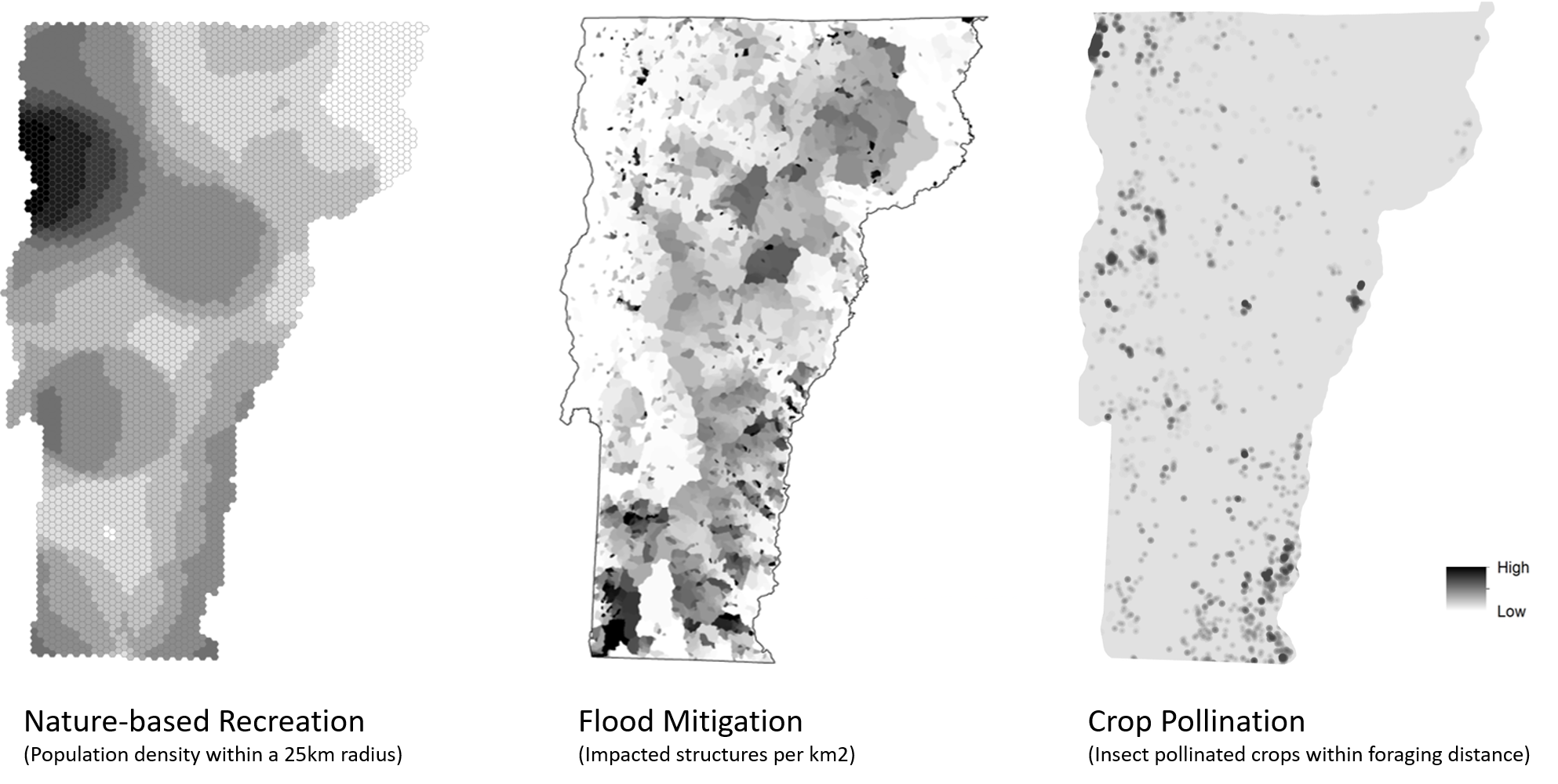


**Appendix S6:** Comparison of biodiversity irreplaceability calculated using BioFinder as a single aggregate measure of biodiversity importance, and using species distributions for the 236 vertebrate species for which distribution data was available in Vermont. Dark areas represent places that are identified as highly irreplaceable: they were included in many of Marxan’s approximations of the optimal conservation network and are therefore likely to be included within the optimal network. Lighter areas were seldom included in best networks, if any, and are thus unlikely to be included in the optimal conservation network.

**
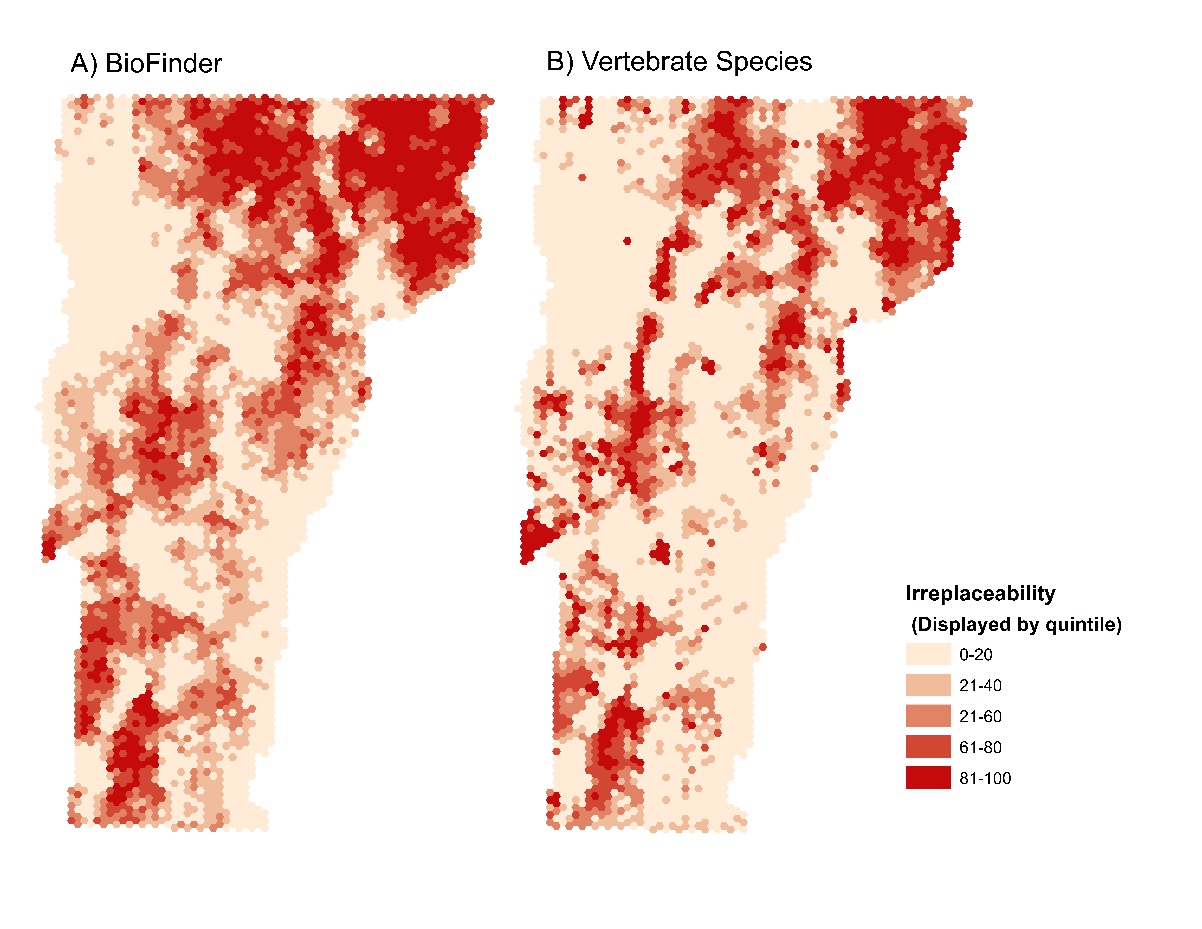
**

**Appendix S7**: Here we compare priority areas identified via single factor Marxan optimizations against priority areas identified as “hotspots” by selecting an equal number of units according to ranked conservation value per unit cost. Areas selected in our single factor Marxan optimizations are shown in pink, hotspots identified by ranked value per unit cost are shown in blue, and areas in common between the two are shown in purple. Percent overlay between these two methods of selecting priority areas for each supply and benefit is shown in the bottom right. Overlay was high on average (75%), and ranged from 43-94%.


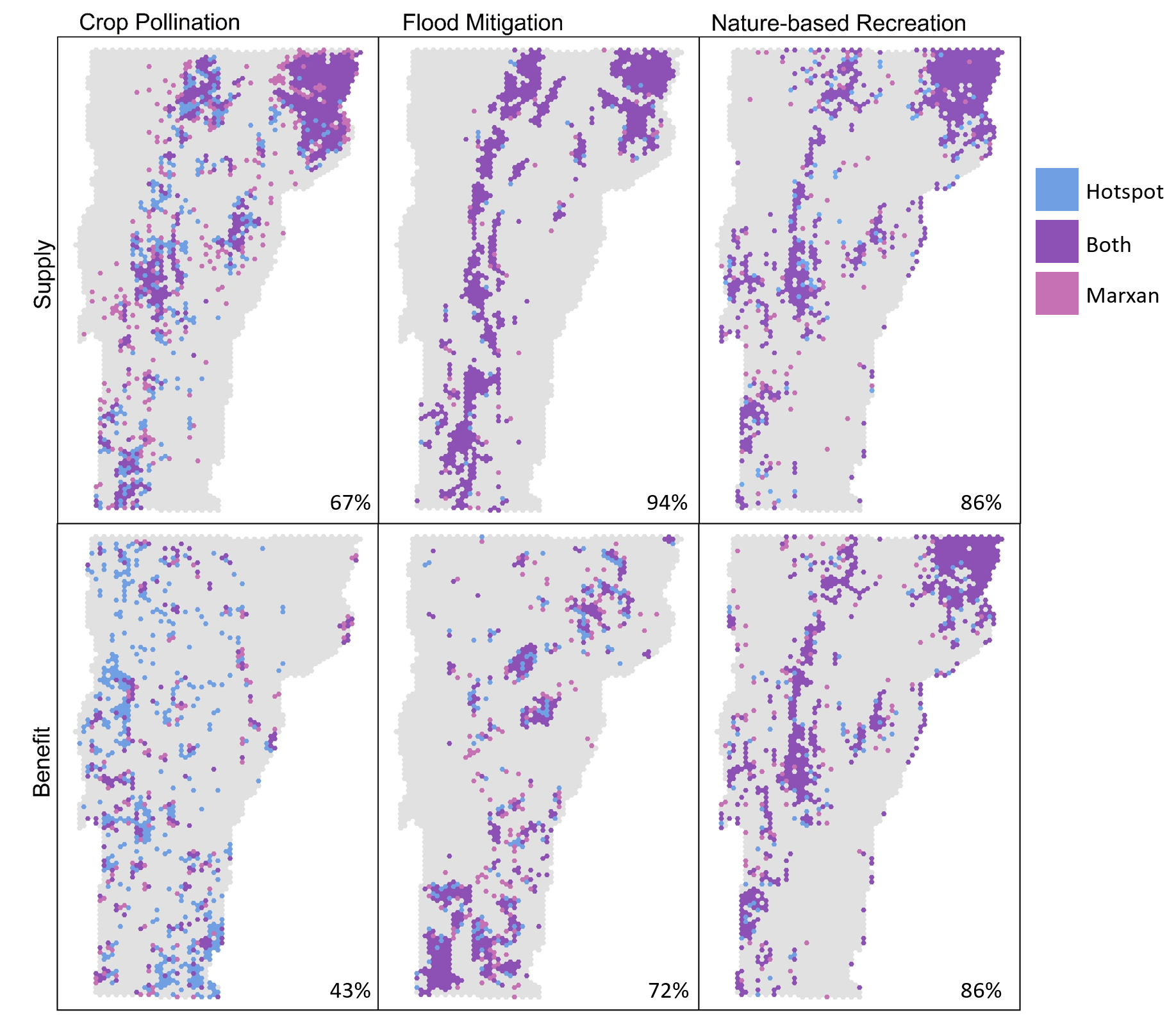

Supplement: Supplementary file 1 — Details on the InVEST Seasonal Water Yield model (Appendix S1), a sensitivity analyses of flood‐mitigation supply results to including winter months in the model (Appendix S2) and of our flood‐mitigation service results to the assumption that supply and demand are equally important in determining benefit (Appendix S3), an explanation of modeled land value as a proxy for conservation cost (Appendix S4), maps of demand for each ES (Appendix S5), a comparison of BioFinder to species‐based conservation prioritization (Appendix S6), and a comparison of single‐factor optimizations to hotpots selected based on ROI (Appendix S7) are available online. The authors are solely responsible for the content and functionality of these materials. Shapefiles of ecosystem service supply, demand, and benefit, and best conservation networks for each are freely available from K.W.’s FigShare account: https://figshare.com/authors/Keri_Watson/6259679. Queries (other than the absence of the material) should be directed to the corresponding author. [file COBI-33-942-s001.docx]
